# The economic impact of eating disorders in children and youth in Canada: a call to action to improve youth eating disorder research and care

**DOI:** 10.1186/s40337-023-00794-z

**Published:** 2023-05-13

**Authors:** Jennifer S. Coelho, Linda Booij, Debra K. Katzman, Gina Dimitropoulos, Nicole Obeid

**Affiliations:** 1 Provincial Specialized Eating Disorders Program for Children and Adolescents, BC Children’s Hospital, Vancouver, BC Canada; 2 Department of Psychiatry, University of British Columbia, Vancouver, BC Canada; 3 Eating Disorders Continuum, Douglas Mental Health University Institute, Montreal, QC Canada; 4 Department of Psychiatry, McGill University, Montreal, QC Canada; 5 Division of Adolescent Medicine, Department of Pediatrics, Hospital for Sick Children, Toronto, ON Canada; 6 Department of Pediatrics, University of Toronto, Toronto, ON Canada; 7 Calgary Eating Disorder Program, Alberta Health Services, Calgary, AB Canada; 8 Department of Psychiatry, University of Calgary, Calgary, AB Canada; 9 Eating Disorders Research Lab, Children’s Hospital of Eastern Ontario Research Institute, 401 Smyth Rd, Ottawa, ON K1H 8L1 Canada; 10 Department of Psychiatry, University of Ottawa, Ottawa, ON Canada

**Keywords:** Eating disorders, COVID-19, Health care utilization, Economic cost analysis, Youth, Health care policy

## Abstract

The COVID-19 pandemic has led to an unprecedented rise in rates and symptoms of eating disorders among Canadian youth. To date, there is a lack of national surveillance and costing data in Canada to inform policymakers and healthcare leaders on how to best address the surge in new and existing cases. This has resulted in the Canadian healthcare system being unprepared to adequately respond to the increased needs. Therefore, clinicians, researchers, policymakers, decision-makers, and community organizations across Canada are collaborating to compare pre-and post-pandemic costing data from national and province-level healthcare systems in an effort to address this gap. Results from this economic cost analysis will be an important first step in informing and guiding policy on possible adaptations to services to better fulfill the needs of youth with eating disorders in Canada. We highlight how gaps in surveillance and costing data can impact the field of eating disorders in an international context.

## Background

Since the start of the pandemic, a surge in eating disorders (EDs) presentations has been reported internationally, including across Canada. Recent public health administrative data have shown that emergency visits and hospitalizations for EDs in children and adolescents in Canada were 1.6 times higher in the first year of the pandemic than between 2017 and 2019, with up to a 132% increase in hospitalization rates [1]. Similarly, data from six pediatric hospitals across Canada indicated a 60% increase in new presentations of restrictive EDs and a near tripling of new cases in the early pandemic as compared to the pre-pandemic [2]. The rise in EDs is alarming since these disorders contribute to significant impairment in mental and physical health and they have among the highest mortality rates of all psychiatric illnesses [3]. The unintended consequences of public health strategies to mitigate the spread of COVID-19 have led to a perfect storm that has magnified a public health crisis in EDs [4].

The pandemic has revealed that an already under-resourced system of care for EDs was unprepared to handle this unprecedented surge in the volume of children and youth with EDs. This can be attributed in part to the lack of surveillance data across the country to accurately identify the number of children and youth with EDs across Canada, leading to policymakers and healthcare leaders being ill-equipped to measure and plan effectively for service needs. It also translates to being unable to quantify the costs and spending occurring to support those with EDs, rendering the system less able to manage when shifts, like the one during the pandemic, occur.

## Economic impact of COVID-19 on eating disorders

Studies related to the prevalence, social and economic costs of EDs have been carried out in the United States (US) [5], United Kingdom (UK) [6] and Australia [7] where national ED organizations and researchers have come together to collectively lead the creation of these reports. The Butterfly Foundation in Australia worked with researchers and community advocates to produce a costing report aimed at improving the understanding of the economic costs of EDs in Australia, in partnership with Deloitte Access Economics [7]. In 2019, the Australian government increased public health funding for ED care [8] and provided funding for a National Eating Disorder Research and Translation Strategy, with the newly created national research centre for EDs (*InsideOut*) responsible for the development of the strategy [9]. Similarly, in the US, the costing study conducted in 2019 generated significant media attention, particularly in outlets catered to policymakers, and resulted in knowledge translation materials that are actively being used in advocating for ED prevention services [10]. Across both studies, findings of the system and personal costs in the billions of dollars have shed light on the need for concerted efforts to address the eating-disorder systems of care. It spoke to the need for national surveillance data to depict costs more accurately across various settings, and for coordinated efforts to drive system change. Neither previous study has examined how the pandemic affected costs nor have they included data from community-based organizations in efforts to round out the usage and costs to the system.

Driven by the lack of available data in Canada to inform the field, and even more compelled by the impact of the pandemic on youth, carers, society and the health system at large, the National Initiative for Eating Disorders (NIED), a parent-led ED advocacy group, ignited conversations with individuals with lived experience, carers, clinicians, researchers, and policy-makers/decision makers to plan a Canadian cost of EDs study, similar to those carried out in Australia and the US. Federal funding from the Canadian Institutes of Health Research (#468607) was obtained by the authors to support a project investigating economic costs related to the pandemic for children and youth with EDs in Canada. This study compares national and province-level healthcare systems’ economic cost data during pandemic times (2020–2022) with pre-pandemic levels in 2019 to understand shifts exacerbated by the pandemic. Survey data and qualitative reports from stakeholders will complement the costing data to help portray a more comprehensive picture of the cost impacts of COVID-19.

It is critical to establish Canadian-specific costing data to inform policy, service allocation, and ultimately system transformation. Developing a comprehensive understanding of the current economic impact will also inform the analysis of the cost-effectiveness of changes in the system (i.e., shifts to virtual care). Within the limited research using Canadian administrative data, gaps in knowledge of ambulatory care for EDs have previously been identified, among other limitations [11]. The present costing analysis allows for obtaining a more precise understanding of the current gaps that are occurring in national data, thereby informing recommendations for improvements in surveillance strategies.

In contrast to the national research institute and ED research strategy that has been developed in Australia, Canadian research on EDs has typically been province-specific and has emanated from provinces with the largest population centres. There is a need to develop national partnerships and collaborations to further knowledge translation and foster cohesive, national collaborations that can respond to priorities that have been identified for ED research in Canada [12]. The economic costing study brings together ED experts from all Canadian provinces, including clinician scientists, decision-makers, individuals with lived experience, and all four national Canadian ED organizations. This effort represents a first step in revolutionizing treatment for EDs in Canada, and includes all members of key stakeholder research groups proposed by Stone and colleagues [13] as a path to use resources more effectively and efficiently. It provides a venue to unify these experts’ voices in efforts to forge change together.

## The path forward: translating research to clinical practice

In working towards effective and efficient use of resources, the first necessary step is the identification of the current costs of EDs in the healthcare system and the impact of shifts in the presentation of EDs in the context of the pandemic. Canadian data suggest that new presentations of EDs have surged with the pandemic [2], suggesting the need for innovative early response services. Similarly the pandemic has also led to increased ED symptoms in individuals who had pre-existing EDs [14], calling on the need to better understand the prevalence of youth with severe and/or enduring EDs and the services accessed by this subsample of youth with EDs. Addressing these surge points without the availability of costing data makes it difficult to identify service gaps and to make recommendations for future service- planning.

Partially based on findings from costing studies performed in other countries are coordinated policy-level responses that have benefitted the ED system of care. For example, in the UK, there is a focus on early intervention, where an evidence-based model, the First Episode Rapid Early Intervention for Eating Disorders (FREED) has been scaled up in over 80% of adult ED services in the country [15]. Similarly, Australia has responded by implementing the FREED model in community-based settings [16], training all primary care mental health providers on ED detection and early treatment, and offering brief interventions to those on waitlists for ED services [17]. The learnings of where costs could be best routed were essential to these shifts.

## Conclusion

Canada is lagging in the development of innovative models of care for EDs in relation to other Western countries. The UK and Australia, for instance, have implemented a service model focused on early intervention, while the Australian government has funded a multi-million-dollar national institute for research, translation and clinical excellence in EDs to execute a strategy to transform ED care across the continuum. Arguably, analyses of the economic costs of EDs in these countries were instrumental steps for the subsequent developments in a national strategy for ED interventions.

Given the context of the current crisis in ED services across Canada, changes to the provincial and national systems of care for people with EDs are urgently needed. Identifying and analyzing available national, provincial, and community-based data to estimate the economic costs of EDs in the healthcare system is an important step to address the scarcity of services for individuals with EDs. It will also give healthcare leaders and policymakers the basis to carry out more precise planning, including budget and resource allocation, as well as adapting existing services for youth with EDs. A recent triangulation approach to inform service planning, based on epidemiological studies, administrative data, and survey data has been proposed to inform needs-based planning [18]. Within an international context, the validity of relying on data derived from treatment-seeking samples has been attributed to misunderstandings within the field (e.g., skewed data for gender or diagnostic presentation) [19]. The need for population-level data across services and levels within the Canadian context is therefore relevant across jurisdictions, to improve the quality and generalizability of research. Finally, this project will allow for international comparisons that will help to identify opportunities to improve care for individuals with EDs and to create a uniform response to prevent another ED crisis in future pandemics or other severe events leading to service disruptions.

## Data Availability

Not applicable.

## Abbreviations

ED: Eating disorder
US: United States
UK: United Kingdom
NIED: National Initiative for Eating Disorders
FREED: First Episode Rapid Early Intervention for Eating Disorders
